# Radiation-Induced Immunity and Toxicities: The Versatility of the cGAS-STING Pathway

**DOI:** 10.3389/fimmu.2021.680503

**Published:** 2021-05-17

**Authors:** Julie Constanzo, Julien Faget, Chiara Ursino, Christophe Badie, Jean-Pierre Pouget

**Affiliations:** ^1^ IRCM, Institut de Recherche en Cancérologie de Montpellier, INSERM U1194, Université de Montpellier, Institut régional du Cancer de Montpellier, Montpellier, France; ^2^ Cancer Mechanisms and Biomarkers Group, Radiation Effects Department, Centre for Radiation, Chemical & Environmental Hazards Public Health England Chilton, Didcot, United Kingdom

**Keywords:** radiation, radiotherapy, targeted radionuclide therapy, inflammation, nucleic acids, bystander immunity, cGAS, STING

## Abstract

In the past decade, radiation therapy (RT) entered the era of personalized medicine, following the striking improvements in radiation delivery and treatment planning optimization, and in the understanding of the cancer response, including the immunological response. The next challenge is to identify the optimal radiation regimen(s) to induce a clinically relevant anti-tumor immunity response. Organs at risks and the tumor microenvironment (e.g. endothelial cells, macrophages and fibroblasts) often limit the radiation regimen effects due to adverse toxicities. Here, we reviewed how RT can modulate the immune response involved in the tumor control and side effects associated with inflammatory processes. Moreover, we discussed the versatile roles of tumor microenvironment components during RT, how the innate immune sensing of RT-induced genotoxicity, through the cGAS-STING pathway, might link the anti-tumor immune response, radiation-induced necrosis and radiation-induced fibrosis, and how a better understanding of the switch between favorable and deleterious events might help to define innovative approaches to increase RT benefits in patients with cancer.

## Introduction

In one century, radiation therapy (RT) has become a cornerstone of cancer treatment and is proposed in about 50% of therapeutic schedules. RT goal is to deliver high amounts of energy in cancer cells that will produce unrepairable damage leading to cell death. However, already the first studies on RT reported that healthy tissues, such as skin, are limiting organs showing specific side effects (for instance, erythema and telangiectasia for skin). The amount of energy delivered to tissues was identified as the critical parameter of RT, and the radiation dose in gray units (Gy) was defined for treatment rationalization. It was also observed that tumors and healthy tissues respond differently when the radiation dose is fractionated. Until the 1940s, various dose and dose per fraction were systemically tested to improve RT efficacy and to better protect skin from early and late reactions (1). This led to the standard therapeutic schedule used today: 2 Gy per fraction, 5 fractions per week, and 6-8 weeks of overall treatment time (2). This has been accompanied by improvements in radiation delivery to the tumor, and the current image-guided radiotherapy systems provide high ballistic precision.

These advances have comforted the target cell theory according to which only tumor cells crossed by radiation will die, ultimately leading to eradication of clonogenic tumor cells and to tumor control. However, exposure of healthy tissues remains a matter of concern (3). Specifically, it has been observed that the response to RT is not the same in all patients, and late radiation toxicities, such as radiation-induced necrosis [RN (4, 5)] and fibrosis (RIF) (6–8), have been described. Besides the intrinsic patient radiosensitivity, total dose, dose per fraction, irradiated volume, and treatment combinations (e.g. endocrine therapy, chemotherapy, history of surgery) (9, 10) could be involved in such side effects.

A new paradigm was established in the 1950s when a possible role for RT-enhanced immune response against cancer cells was suggested. Regression of cancer cells at a distance from the radiation field was reported, leading to the introduction of the *abscopal* effect concept (11). These observations that challenge the target cell theory have been supported by many other studies (12–15), and the immune response role during RT is today strengthened by the benefit observed when combining RT and immunotherapy, which stimulates or suppresses the immune system to help the body fight cancer (e.g. monoclonal antibodies) (14, 16, 17).

Here, we will review how RT modulates the immune response towards a better tumor control or side effects associated with inflammatory processes. After briefly describing the cellular and tissue responses to RT and the different RT modalities, we will discuss how the innate immune sensing of RT-induced genotoxicity might link anti-tumor immune response, RN and RIF, and how a better understanding of the switch between favorable and deleterious events might help to define innovative approaches to increase RT benefit in patients with cancer.

### Cellular and Tissue Responses to RT

RT is based on the principle that radiation will produce lethal lesions in exposed cells. This starts with the ionization and excitation of molecules contained in cells, leading to the production of radical species, such as reactive oxygen (ROS) and nitrogen species (NOS) that will damage cell constituents. These damages may be repaired (cells will survive), misrepaired (cells undergo abnormal proliferation), or not repairable (cells will die). Among all the radiation-sensitive targets, nuclear DNA has been the most investigated. Indeed, survival of irradiated cells is closely related to the level of unrepaired DNA double-strand breaks, and the DNA damage response (DDR) plays a major role in the final cellular outcome. Other subcellular targets, such as cell membrane (18–20), mitochondria (21, 22) and lysosomes, also may contribute to the final outcome. It must be noted that cell killing will be more important when the dose and dose-rate increase than when the dose is fractionated or delivered at low dose-rate.

Target cell death upon RT leads to reduction in tissue function (1). As RT delivers high fractionated dose (2 Gy per fraction, 5 fractions per week, total dose between 40 and 70 Gy), the priority is to precisely control the exposure to radiation of tumor cells and healthy tissues. The determinist effects, occurring beyond a certain dose-threshold (>0.5 Gy), are proportional to the dose, according to a *S* shape curve (sigmoid curve), before reaching a plateau at high dose. Therefore, by controlling the dose, it is possible to predict the biological effect, e.g. the tumor control probability. The S curve obtained for healthy tissues (normal tissue complications probability) is quite similar as the one obtained for tumor cells, but the dose threshold is higher. This indicates that the tumor is more sensitive to radiation than healthy tissues when using the previously described fractionated schedule. Therefore, it is possible to define a therapeutic window where tumor growth can be controlled with acceptable side effects. The organs concerned by deterministic effects usually display high proliferation rates (i.e. tumor, skin, bone marrow, digestive tract), but other organs also may be concerned, for instance the nervous system.

### RT Modalities and Differential Effects on Tissues

It took more than 50 years of preclinical and clinical data to define the current standard therapeutic schedule of RT. This schedule allows controlling the tumor, while minimizing side effects. At the beginning of RT, the first systems produced low energy X-rays that delivered huge doses to the skin, which was used as the guide for therapeutic schedules. Schedules were progressively improved to deliver the maximum dose not to the skin but to the tumor. This was the beginning of a huge progress in the design/development of technological devices with the final goal of increasing the ballistic accuracy and improving the ratio between disease control and toxicity (23). Three-dimensional conformal radiation therapy (3D-CRT), intensity-modulated radiation therapy (IMRT), stereotactic body radiation therapy (SBRT/SABR) and stereotactic radiation surgery (SRS), proton therapy (and to a lesser extent hadrontherapy with heavy ions) (24), and more recently FLASH RT (25) have progressively been implemented. For example, 3D-CRT aims at delivering radiation to the gross tumor volume with a margin for microscopic tumor extension and a further margin uncertainties for organ in motion, while IMRT allows the oncologist create irregular-shaped radiation doses that conform to the tumor whilst avoiding critical organs. For instance, the optimal radiation technique to treat breast cancer may vary with patient anatomy and laterality of the breast cancer. IMRT provide better conformality of the high dose to the target regions than conventional 3D-CRT, but at the expense of more tissue (contralateral breast and lung) exposed to low radiation doses. Also, due to physical properties, proton therapy improves target coverage and conformality with a high dose volume to the target, and significantly reduces both organs at risks and integral doses. Thus, the more the radiation technique allows a perfect coverage of the tumor shape while avoiding healthy surrounding tissues, the more the dose can be increased (improving the cytotoxic effect of the physical dose), intensificated, or hypofractionated to further improving outcomes.

However, some of conventional RT modalities are not always suitable for the treatment of disseminated or diffuse disease or of tumors located very close to organs at risk because it would lead to an unacceptable exposure of healthy tissues to high radiation doses. Very early, clinical radionuclides were identified as an alternative to RT because they emit radiation and can be used as unsealed sources for intravenous injection. In 1941, iodine 131 (26), which is taken up by the thyroid gland, was the first tested radionuclide for hyperthyroidism treatment, marking the birth of nuclear medicine (27). Recently, Xofigo™ (^223^RaCl_2_) has been approved for bone metastasis management in patients with prostate cancer (28). In brachytherapy (also called Curietherapy), radionuclides are locked in a sealed capsule placed close to the tumor (e.g. prostate cancer), and then the radiations cross the capsule and irradiate the localized tumor. In 1951, for the first time, radionuclides were radiolabeled with vehicles, such as monoclonal antibodies against cancer cells (29–31) and later peptides. For instance, Lutathera™ (^177^Lu-DOTATATE) has been approved for treating neuroendocrine tumors (32–34). However, radionuclide therapy also is associated with side effects due to exposure of healthy tissues. For example, treatment with Lutathera™ strongly increases progression-free survival in patients (32), but whole blood and bone marrow are inevitably exposed to radiation that may lead to long-term toxicities. Subacute hematologic toxicity (grade 3/4) after Lutathera™ has been observed in 11% of patients (35), and long-term safety concerns include myelodysplastic syndrome (MDS) and leukemia (32, 36).

The choice between the different RT modalities depends on the tumor type and its localization. The chosen modality will influence the delivered dose and dose-rate and the nature of the lesions produced in cells. For example SBRT and SRS, which deliver high individual radiation doses with enhanced precision accuracy in only few treatment fractions, can be used to ablate small and well-defined primary tumors anywhere in the body, such as non-small cell lung cancer (NSCLC) (37–39), or brain metastases (SRS) (40, 41). However, these modalities may cause late RIF and RN. RN is a well-characterized effect of SRS and is occasionally associated with serious neurologic sequelae (42). A preclinical study in normal rats whose brain was exposed to a single radiation dose (37 Gy at 30% using a Gamma Knife^©^ device) found vascular disorders and neovascularization (43) with no detectable behavior changes at day 54 post-irradiation. At day 110, rats exhibited large RN surrounded by an increasing gradient (distal to proximal from the RN) of microglia that accumulated near newly sprouted blood vessels, upregulation of Iba1^+^CD68^+^ macrophages, and infiltrating CD3^+^ T cells (44). These effects were accompanied by irreversible neuroinflammation, memory loss and a decrease in anxiety-like behavior (44). In the context of brain RN pathophysiology, there are two main theories whether it is likely that the true cause is multifactorial: i) the vascular injury theory and ii) the glial cell theory. In the first case scenario, radiation disrupts the blood-brain barrier, resulting in increased capillary leakiness and vascular permeability. Radiation, especially in large fraction sizes >8 Gy, activates acid sphingomyelinase and causes upregulation of ceramide, which in turn causes endothelial apoptosis (20, 45). This leads to increased oxygen-free radicals, a pro-inflammatory milieu (through release of tumor-necrosis factor and interleukin-1β) (46), and amongst other increased production of vascular-endothelial growth factor (VEGF). This cascade leads to anarchic vessel sprouting resulting in ischemia and cell death (47). In the second case, radiation can also damage glial cells. Damage to oligodendrocytes and their progenitors result in demyelination (48), accompanied by leaky capillaries, which result in perilesional edema (43, 48). Therefore, it is important to understand the balance between beneficial and deleterious effects of the radiation-induced inflammatory response, and how exposed tumor cells communicate with their microenvironment.

### Revisiting the Target Cell Paradigm Accounting for Non-Irradiated Bystander Cell Killing

For about one century, RT has been considered as a ballistic therapeutic approach where radiation is seen as projectiles targeting tumor cells. Accordingly, only cells traversed by radiation will die. There is now a huge body of evidence indicating that irradiated cells communicate with non-irradiated neighboring cells, leading to the so-called bystander response to radiation that includes cytotoxic and genotoxic effects, such as chromatid exchange (49), mutagenic effects (50), micronucleus formation (51) and DNA damage-inducible protein upregulation (52, 53). Besides these short-distance effects, there are long-range effects that involve the immune response activation through the production/release by irradiated cells of pro-immunogenic factors, such as tumor antigens (54), Natural Killer (NK) receptor G2D (NKG2D) ligands that act as danger signals to alert NK cells (55), and through the recruitment of CD8^+^ T cells and myeloid cells (56) together with the production of type I Interferon (IFN) (57). Simultaneously, RT can lead to immunogenic death of cancer cells (15) that can subsequently favor the immune cell response toward the surveillance and eradication of transformed cells (58). Immunogenic cell death consists in the release of immunostimulatory damage-associated molecular patterns (DAMPs) by dying cells (59), for instance extracellular ATP (60), extracellular DNA (61), nuclear DNA-binding protein high mobility group box 1 (HMGB1) (62), and endoplasmic reticulum chaperones, such as calreticulin (63). Irradiated cells produce also inflammation-related cytokines (e.g. IFNs, IL-1, IL-6, IL-8, VEGF, EGFR, and TNFα) encoded by ‘‘early response’’ genes (64) that are induced within minutes to hours following RT exposure. This is associated with ROS production and cytokine production that will participate in the creation of a DAMP-associated proinflammatory micro-environment. Mediators of systemic effects and DDR/DNA repair components interact also with components of the innate immune response, such as pattern recognition receptors, and with DNA repair proteins (BRCA1, XRCC1, DNA-dependent protein kinases, Ku70/80) (64). For instance, during RT (or chemotherapy), dendritic cells (DCs) require signaling through Toll-like receptor 4 (TLR4) for efficient processing and cross-presentation of antigen from dying tumor cells (releasing HMGB1). Apetoh et al. demonstrated that *in vivo*, local RT reduced tumor growth on CT26 colon cancers and TS/A breast carcinomas, and prolonged the survival of tumor-bearing immunocompetent wild-type mice, which was less effective in Tlr4^-/-^ and athymic nude mice (65).

### Critical Tissues and Cell Response to RT: Bone Marrow and Circulating Blood Cells

Although treatment planning allows delivering most of the radiation dose to the tumor, the surrounding healthy tissues also are exposed to radiation, but at lower doses. Consequently, the surrounding tissues, including the vascular system, also are included in the exposed volume. The tumor and infiltrating immune cells (myeloid cells and lymphocytes), whose number depends on the tumor immune microenvironment (hot, cold, and immune-altered), are also irradiated. Consequently, RT can have detrimental effects on the hematological compartment. Bone marrow aplasia occurs for doses >3 Gy and death due to hematopoietic syndrome occurs upon whole-body exposure to doses that are expected to cause the death of 50% of exposed people (LD50 = 4.5 Gy) (66). When irradiation is not fatal, the number of hematopoietic stem cells returns progressively to normal, but this can take years. Higher intramedullary cytotoxicity due to abnormal hematopoiesis can be observed, although blood formula has returned to normal values. This might be due to RT-linked modifications of the stem cell microenvironment, niches and/or vascularization. Long term effects of irradiation of bone marrow have been reported in patients treated for ankylosing spondylitis (67) or in atomic bomb survivors. They mainly consist of acute leukemia a myelodysplasia occurring between 5 and 10 years after exposure. However their occurrence depend on the dose and have not been observed after RT alone but more after combination with chemotherapy (68, 69).

Bone marrow is a tissue with a hierarchical organization that is involved in the early response to RT. Quiescent or proliferating hematopoietic stem cells are located in bone marrow. Except for T lymphocytes that differentiate in thymus, hematopoietic cells proliferate and differentiate in the bone marrow before entering the blood circulation. During RT, a proportion of stem cells is killed and the negative effect on hematopoiesis is proportional to the irradiation dose. As blood cells have a limited lifespan, blood cell depletion will be detectable after the non-replacement of mature cells by young differentiating cells. The immune cell radiosensitivity depends on the lineage, maturity, and activation status. All bone marrow cells and particularly progenitors are sensitive to RT, and 1 Gy kills about 2/3 of all progenitor cells. Conversely, mature cells, except lymphocytes, are relatively resistant to RT. Lymphocytes are particularly radiation-sensitive, and a decrease in circulating lymphocytes, due to apoptosis, is observed already with 0.3 Gy. At 1 Gy, the decrease becomes significant and occurs within 3 days. B cells and naïve T helper (Th) cells are the most radiation-sensitive, whereas T memory cells, natural killer T cells, and regulatory T cells (Tregs) are more resistant (64).

In recent years, the transcriptional response to radiation exposure has been much studied. This is important because it has been reported that the gene expression signature of blood lymphocytes can help to predict the clinical outcome in human cancers (70). Upon exposure to RT, multiple signal transduction pathways are activated, resulting in complex alterations in gene expression in circulating immune cells [e.g. Kabacik et al. (71)]. For instance, CD4^+^ and CD8^+^ T cells produce IFN-γ, contributing to the formation of an inflammatory environment that favors the anti-tumor immunity (72). Irradiated human monocytes and macrophages activate transiently p53- and ATM-dependent mechanisms. The transcriptional factors TP53 and nuclear factor kappa B (NF-κB), which play a central in immune and inflammatory responses by regulating the expression of pro-inflammatory cytokines and chemokines such as TNF-α, induce the expression of inflammatory cytokine-encoding genes, thus establishing a direct link between radiation-induced DNA damage response and radiation-induced inflammation (73).

Circulating leukocytes are only exposed when passing through exposed blood vessels and receive a much lower dose, which is difficult to calculate accurately. Yet, this is a crucial issue because the transcriptional changes observed *in vitro* following exposure of whole blood samples are quantitatively (74) and qualitatively (75) different in function of the dose. High doses induce mainly p53-dependent signaling, and genes involved in the stress response and apoptosis. Their level of expression is dose-dependent down to 10-50 mGy. Low doses predominantly induce the NF-κB pathway and the regulation of genes involved, for instance, in cytosolic DNA sensing and chemokine and cytokine signaling, rather than radiation-induced direct cell killing. NF-κB, p53, breast cancer associated protein 1 (BRCA1) and AP-1 are among the main transcription factors activated by radiation exposure and regulated by the DDR (76), but low doses induce more immune-stimulatory responses (75). Therefore, it can be hypothesized that the dose received by immune cells and consequently the triggered responses are determined by their localization during RT. Interestingly, the influence of the tumor presence on the expression of several stress genes in circulating white blood cells has been investigated, and similar levels of expression in pre-exposure cancer samples and in normal donor samples were observed (77).

Additionally, the type of radiation (X-rays, gamma, proton, beta or alpha particles), the dose rate (around 1 Gy per minute, FLASH irradiation in seconds, or protracted - days - irradiation in targeted radiotherapy, TRT) and the RT type (e.g. IMRT or SABR), which limits the dose to the microenvironment and surrounding organs, can modify the volume of irradiated blood, the dose to circulating leukocytes and consequently the associated transcriptional modifications. This is illustrated by the different modulation of the expression of inflammation genes, such as TGFβ1 (cytokine with anti-inflammatory properties), IL-1β and IL-6 (pro-inflammatory), CCL3 (involved in the recruitment and activation of granulocytes) and IL8 (neutrophil recruitment), in function of the RT type (IMRT and SABR) and total dose (78). For instance, TGFβ may be a major obstacle to the optimal activation of antitumor T-cell responses by RT. Bouquet et al. demonstrated that TGFβ inhibition prior to radiation attenuated DNA damage responses, increased clonogenic cell death, and promoted tumor growth delay, and thus may be an effective additional therapy in cancer RT (79). Also, in preclinical models of metastatic breast cancer, Vanpouille-Box et al. showed that anti-TGFβ antibodies administered during RT uncovered the ability of RT to induce T-cell responses to endogenous tumor antigens (80). Interestingly, only the combination of RT with anti-TGFβ, but not each treatment alone, induced T-cell-mediated rejection of the irradiated tumor and non-irradiated metastases in mice, indicating that blocking TGFβ unleashes the potential of RT to promote an *in situ* tumor vaccine (80). In addition, TGFβ activation depends on radiation modalities. Vozenin’s research team demonstrated that conventional RT (15 Gy) triggered lung fibrosis associated with activation of the TGFβ cascade, whereas no complications have been observed after doses of FLASH below 20 Gy for more than 36 weeks after irradiation (81).

Also, the effects of RT on suppressive immune cells, such as regulatory T cells (Tregs), in the tumor microenvironment (TME) are not fully elucidated. For example, across several tumor models (B16/F10, RENCA, and MC38) Muroyama et al. demonstrated that RT (10 Gy) significantly increased tumor-infiltrating Tregs compared with non-irradiated tumors. The authors found that tumor-infiltrating Tregs from irradiated tumors had equal or improved suppressive capacity compared with non-irradiated tumors, independently of TGFβ (82). Consequently, blocking Tregs infiltration in tumors might be an interesting therapeutic strategy in combination with RT and anti-PD-L1, to overcome RT-induced immunosuppressive Tregs and drive an abscopal effect (83).

In conclusion, there is a direct link between radiation-induced DNA damage-dependent changes in gene expression and radiation-induced inflammation. These changes need to be better investigated to decipher these complex interactions.

Overall, this section showed the complex interaction between ionizing radiation, tumor cells and TME. It also highlighted that not all observed effects are linked to direct radiation damage crossing cancer cells, but also to bystander and systemic effects.

## Janus-Faced Tumor Microenvironment Components During RT

RT is detrimental for bone marrow and circulating blood cells through their direct irradiation, but it can also *via* its indirect effects, trigger the activation of immune cells, as observed when RT is combined with immunotherapy (84). RT physical parameters, such as dose and dose-rate, are key determinant of the response type. Nevertheless, it must be kept in mind that the immune response can participate in cancer control, but can also contribute to the deleterious inflammatory effects observed in healthy tissues. The balance between radiation-induced immunity and toxicity is influenced by the TME cell composition, architecture and intercellular communications. The role of macrophages, endothelial cells, fibroblasts and mesenchymal stem cells (MSC) in the TME is presented in the following paragraphs.

### Macrophages

In macrophages, ionizing radiation induces the pro-inflammatory phenotype that favors their pro-invasive and pro-angiogenic functions *in vitro* (85). This involves the transient activation of p53- and ATM-dependent responses. The transcription factors p53 and NF-κB, which have key roles in the immune and inflammatory responses, regulate the expression of pro-inflammatory cytokines and chemokines, such as TNF-α, and lead to the expression of inflammatory cytokine-encoding genes, thus establishing a direct link between radiation-induced DDR and radiation-induced inflammation (73). Indeed, Mikhalkevich et al. demonstrated macrophages irradiation induced an altered secretory phenotype (through human endogenous retroviruses), characterized by an increase of proinflammatory factors, such as IL-6, IL-1β, TNFα, CCL2, CCL3, CCL8, and CCL20, in addition to an elevated secretion of anti-inflammatory IL-10, which may facilitate their tumorigenic activity (86). In mice xenografted with insulinoma, melanoma or prostate cancer cells and exposed to low radiation doses (2 Gy), macrophages in the TME show increased inducible nitric oxide synthase (iNOS) expression that favors their ability to inhibit abnormal tumor angiogenesis and promote tumor antigen-specific T-cell immunity (87). The activation of a signaling cascade involving NOX2-mediated ROS production, ATM and IRF5 is required in 2 Gy-irradiated macrophages for the acquisition of the RT induced pro-inflammatory phenotype. Moreover, NOS2^+^CD68^+^ macrophages are enriched in tumor lesions from patients with colorectal cancer showing good response to neoadjuvant RT (88). Interestingly, a study based on the observation that human papillomavirus 16 (HPV16)-positive head and neck cancers are more sensitive to immunotherapy than HPV16^-^ specimens found that IL-6 production by HPV16^+^ cancer cells specifically favors RT-induced macrophage polarization toward an immunostimulatory phenotype, which is linked to the establishment of an effective anti-tumor immunity (89). Furthermore, blockade of IL4/IL14 signaling by inhibiting STAT6 suppresses the induction of the immunosuppressive phenotype in the THP1 human macrophage cell line, thus reducing the radiation resistance of the co-cultured inflammatory breast cancer cell lines (90). Macrophage behavior following radiation appears versatile and influenced by the TME. However, 2 Gy irradiation of mouse macrophages reduces their ability to induce T-cell proliferation *in vitro* (91, 92). The positive impact of macrophages following RT remains largely debated because despite the induction of a pro-inflammatory phenotype, these cells are unfavorable to the establishment of an effective anti-tumor immune response in multiple contexts. In agreement, macrophage depletion upon 10 Gy RT promotes the adaptive immunity and the response to immune checkpoint inhibitors in mice harboring MC38 colorectal cancer cell xenografts (93). Similarly, 25 Gy irradiation and 4 Gy fractionated irradiation of mice xenografted with TRAMP-C1 prostatic cancer cells drive ARG1, iNOS and COX2 expression in macrophages. Moreover, the transfer of macrophages isolated from 25 Gy-irradiated tumors increases tumor growth *in vivo* (94). Finally, CD163 expression, a marker of immunosuppressive macrophages, is negatively associated with survival in patients with HPV16^-^ head and neck primary tumors after RT with various radiation modalities (95).

Although macrophage plasticity in response to the radiation modalities and TME might favor the anti-tumor immune response and radiation resistance, this cell type has been constantly associated with RT-induced toxicity. In mice exposed to localized colon irradiation, depletion of monocytes and macrophages using clodronate is associated with a major reduction of colon infiltration by T lymphocytes, an 1.4-fold decrease of colon vascularization and lower collagen deposition in crypts, suggesting a reduction of the fibrotic process (96). In mice, irradiation of the upper region of the right lung (20 Gy as single dose or fractionated) leads rapidly (72 hours) to infiltration by macrophages and neutrophils and later to collagen deposition and fibrosis (week 26) (97). Interestingly, in mice, soy isoflavones increase Arg1^+^ immunosuppressive macrophage survival, avoid immunostimulatory phenotype activation in interstitial macrophages, and reduce neutrophil recruitment following 10 Gy irradiation to the lung (98). Similarly, treating mice with the anti-inflammatory fucoidan reduces the accumulation of macrophages and neutrophils after 10 Gy irradiation that is associated with decreased expression of CXCL1, TIMP1, MCP1 and MIT2 (99). These modifications in the early response to RT are particularly important because lung fibrosis was strongly decreased in this model. Co-inhibition of PDGF and TGFβ in mice during and after lung irradiation (20 Gy) strongly reduces lung fibrosis and increases mouse survival. Similar results and the concomitant reduction of immunosuppressive macrophage infiltration in lungs were obtained by blocking connective tissue growth factor (CTGF) in mice (100, 101). CTGF blockade might abrogate TGFβ downstream effects (cell mobility and epithelial-to-mesenchymal transition, EMT) on MSCs, fibroblasts and endothelial cells (101), and deeply remodels the lung immune infiltration following RT (102). In a rat model of RT-induced gut toxicity, 25 Gy irradiation of the gut led to increased expression of MMP2, MMP9, VEGF, TGFβ, endostatin and angiostatin. These factors might strongly influence the behavior of endothelial cells (103). In conclusion, most cellular responses associated with lung fibrosis are caused by or linked to infiltration by macrophages with a pro-inflammatory phenotype.

### Endothelial Cells

The establishment of an effective anti-tumor immune response depends on the functionality of the tumor vasculature. Yet, ionizing radiation profoundly modifies blood vessel functionality by activating ATM signaling, oxidative stress responses and DAMP signaling in endothelial cells that ultimately drive NRF2, AP-1 and NF-κB activation [for review see Baselet et al. (104)]. Interestingly, genetic engineering allows the specific sensitization to RT of the vasculature or of tumor cells through the conditional knockout of the *Atm* gene in cancer or endothelial cells in a mouse model of lung adenocarcinoma. Strikingly, RT anti-tumor activity is not increased in mice where *Atm* was knocked out specifically in endothelial cells, despite the massive destruction of the tumor vasculature. Conversely, *Atm* knockout specifically in cancer cells strongly increases the response to RT (105). Hence, in some RT modalities, endothelial cells can be killed by radiation, but this does not seems to contribute significantly to the sensitivity to RT. It is noteworthy that regarding ATM signaling in a tumor context, Zhang et al. demonstrated that ATM regulates IFN signaling in pancreatic cancer such that its inhibition induces TBK1 activation and IFN-I production that is further enhanced by RT (106). *In vivo*, the authors showed that ATM silencing increased IFN signaling as well as PD-L1 expression. Consequently, ATM-deficient tumors are sensitized to combination therapy with PD-L1 blockade and RT. The regulation of IFN signaling by ATM represents a connection between the radiation-induced DDR and innate immunity that can be exploited to enhance the efficacy of immune checkpoint blockade therapy.

Exposure of human coronary artery endothelial cells to 10 Gy irradiation (single dose or five fractions of 2 Gy) leads to higher modifications of the DDR, immune response, apoptosis and inflammatory response gene expression profile upon fractionated treatment. DDR and the expression of DNA repair genes were decreased in irradiated cells, while expression of ICAM1, VCAM1, CXCL10, CXCL11, CXCL12, CXCL16, CCL2, CCL5, CCL20, CCL23, IFNE, IFNA4, IL1A, IL1B, IL15, TGFB1, TGFB1, CXC4, CXCR7 and FAS was increased (107). In TNFα pre-activated endothelial cells, exposure to low radiation doses (0.3 to 0.6 Gy) reduces leukocyte adhesion, unlike moderate doses (2-5Gy). This suggests that differences in radiation doses might confer to endothelial cells the capacity to support (<2Gy) or reduce (<0.5Gy) immune cell extravasation (108). Similarly, 2-6 Gy irradiation of endothelial cells increases cancer cell/endothelial cell adhesion *in vitro*, and this effect is enhanced by pre-incubation with TNFα. Furthermore exposure of human umbilical vein endothelial cells (HUVEC) to 2 or 4 Gy photon irradiation increases the endothelial cell monolayer permeability for tumor cells through a mechanism involving ADAM10-mediated degradation of VE-cadherin (109). Thus, through the induction of an inflammatory response, radiation reduces the endothelial barrier permeability and promotes the release of pro-inflammatory factors that orchestrate the architecture of the tumor immune microenvironment. The exact contribution of endothelial cells to the induction or the suppression of an effective anti-tumor immune response upon RT remains unclear. Nevertheless, the implication of these cells in RT-induced cardiac toxicity is well established through the induction of cell death, premature senescence and pro-thrombotic reactions (110, 111). Moreover, deletion of plasminogen activator inhibitor type-1 in endothelial cells protects mice from RT-induced colitis through a reduction of macrophage accumulation and collagen deposition in the irradiated colon (112). Similarly, inhibition of radiation-induced CCL2 signaling preserves lung endothelial cell function in irradiated mice, reduces macrophage and neutrophil contribution to lung fibrosis, and metastatic colonization (113).

### Fibroblasts and Mesenchymal Stem Cells

After cancer cells, fibroblasts are the main cell population in the TME of many solid cancers. They play a crucial role in the TME and cancer progression, and they are usually referred to as cancer-associated fibroblasts (CAFs). CAFs are considered to be extremely resistant to RT, and indeed they are not killed by exposure to high radiation doses (18 Gy) (114, 115). Fibroblasts are normally in a resting state with low transcriptional and metabolic levels, but they can change to a more active phenotype following RT. Once activated, fibroblasts start to produce and secrete many factors, such as cytokines, ROS, nitric oxide (NO) and extracellular matrix components (116), that strongly influence the TME effects on immune and cancer cells. CAFs have been extensively described as suppressor cells for both innate and adaptive immune responses. After a single dose (18 Gy) or fractionate irradiation (3 x 6 Gy), CAFs can inhibit the migratory capacity and pro-inflammatory cytokine secretion of immunostimulatory macrophages, redirecting them toward an immunosuppressive phenotype (117). RT-treated CAFs (1 x 18 Gy or 4 x 2 Gy) also suppress Tcell function and migration through the secretion of soluble factors that inhibit IFNγ and TNFα production by T cells (114).

The CAF secretome after irradiation influences also cancer cells behavior. Upon activation induced by irradiation (1.8, 9, or 18 Gy), CAFs isolated from human colorectal cancers secrete IGF1 that then activates the mTOR pathway in cancer cells, thus promoting their survival and proliferation, especially at high radiation dose (115). Similarly, in a model of pancreatic ductal adenocarcinoma, conditioned medium from irradiated fibroblasts (5 Gy) increases iNOS/NO signaling in cancer cells, activating the production of pro-inflammatory cytokines through NF-kB signaling. The activation of this pathway increases cancer cell aggressiveness, with higher cell growth, migration invasion and metastatic potential (118). CAFs promote cancer cell aggressiveness also by secreting factors that induce EMT. For instance, upon exposure to 4 Gy, CAFs secrete CXCL12 and IL-6 that drive EMT in pancreatic cancer cells, making them more prone to migration and invasion (119). Also, RT-induced-CAF-dependent IL-6 expression plays a crucial role in EMT of esophageal adenocarcinoma cells, as shown by monitoring the effect of conditioned medium of fibroblasts isolated from patients after treatment (chemotherapy and radiotherapy) (120). This CAF-dependent mesenchymal phenotype is also associated with resistance to radiotherapy (120). Most importantly, CAFs influence the TME also by remodeling its structure (121) through the production of collagen, fibronectin and other extracellular matrix (ECM) components (122). Following RT, this process is accompanied by downregulation of metalloproteinase expression and culminates in the accumulation of ECM components. ECM restructuration and the pro-inflammatory and highly oxidative microenvironment created by CAFs can lead to tissue fibrosis (121)

A promising approach to overcome RIF is based on the use of MSCs (123). MSCs migrate to the injured tissue also thanks to the expression of CXCR1 that binds to IL-8 produced by RT-damaged cancer cells (124, 125). There, they can regenerate the damaged tissue through their ability to differentiate into various cell types. Several evidences highlight MSC important contribution to RT-induced vascular injury repair by differentiating into endothelial cells (124, 126). MSC role in RIF repair is also mediated by their immunomodulatory secretome that counteracts inflammation and oxidative stress in fibrotic tissue caused by CAFs and cancer cells (127, 128). Inhibition of RT-derived inflammation by MSCs also decreases the risk of lung metastases after irradiation (124). Moreover, in a mouse model of melanoma, the response to RT (2 Gy) is enhanced by associating local or systemic injection of MSCs (129). Similar results were obtained in a mouse model of irradiated glioblastoma (10 Gy) (125). Hence, MSC administration appear to be a key strategy to counteract RT side effects and improve its outcome.

Altogether, these observations highlight that common mechanisms are involved in RT-induced anti-cancer immunity and side effects. Indeed, the amplification of the anti-tumor immunity and deleterious fibrosis and necrosis are the consequence of bystander transmission of ROS-induced cell stress through macrophages, endothelial cells, fibroblasts and MSC sterile-inflammatory responses. A new component, called STING-mediated innate immune signaling, has recently be added to this complex anti-cancer immunity-side effects cross-talk. Accumulating evidences tend to position this pathway at the interface between RT-induced immunity and toxicity.

## The STING Pathway in RT Induced Immunity

To detect pathogens, the mammalian innate immune system has evolved distinct sensing strategies, including extranuclear DNA recognition. Nucleic acid-sensing is based on cytosolic receptors that detect extranuclear DNA or extracellular RNA as DAMP signals. These pathways can trigger cell death in malignant cells and recruit immune cells into the TME, and are investigated as promising adjuvants in cancer immunotherapies (130). To date, one of the major pathways that mediate the immune response to DNA is governed by the DNA-sensing enzyme cyclic guanosine monophosphate–adenosine monophosphate (cyclic GMP–AMP) synthase (cGAS) (131, 132). cGAS is activated upon binding to double-stranded DNA (dsDNA). Activated cGAS converts adenosine 5´-triphosphate (ATP) and guanosine 5´-triphosphate (GTP) into cyclic GMP–AMP (cGAMP). Cyclic GAMP acts as a secondary messenger that binds to and activates stimulator of interferon genes (STING), ultimately triggering a variety of inflammatory effector responses (133). In addition, retinoic acid inducible gene-I (RIG-I) and melanoma differentiation-associated protein 5 (MDA5) might induce growth inhibition or apoptosis of different cancer cell types upon activation by RNA ligands in an IFN-dependent or -independent manner (134). This review focuses only on the cGAS-STING pathway.

### Radiation Induces Cytosolic Double-Stranded DNA Accumulation That Is Sensed by the cGAS-STING Pathway

Radiation-induced chromosomal aberrations represent an early marker of late effects, including cell killing and transformation (135). Micronuclei are small nuclei found in the cytoplasm in addition to the primary cell nucleus of mammalian cells and are produced during mitosis by various mechanisms (e.g. acentric fragments, multicentric chromosomes, etc.) (136). When damaged cells go through mitosis, micronuclei may follow four major possible fates: degradation, reincorporation, extrusion, and persistence (137). Micronuclei may be degraded in the cytoplasm after collapse of their nuclear envelope, leading to irreversible loss of compartmentalization during interphase, and are characterized by chromatin compaction (138). Hatch and colleagues observed multiple foci or a single large focus of accumulated γ-H2AX in approximately 60% of disrupted micronuclei located in the cytosol of cancer cells, indicating that DNA damage accumulation is strongly correlated with micronucleus disruption (138). In the context of ionizing radiation, micronucleus production increases in function of the irradiation dose (139) and is correlated with cell killing. Moreover, Piron et al. demonstrated that mis- or un-repaired DNA double strand breaks might lead to micronucleus formation and to mitotic death of damaged cells (140). However, these data suggest that acute cell death associated with low doses and low dose-rate of ^125^I-labeled antibodies (Auger electron emitters) is not due to defective detection of DNA damage by the cells. Impaired repair of double strand breaks might be involved in the low dose-rate efficacy of TRT using ^125^I-labeled antibodies in a non-dependent dose-effect relationship (140).

Accumulation of dsDNA in disrupted micronuclei present in the cell cytosol can explain the activation of the cGAS-STING pathway following RT (141). When the nuclear envelope of a micronucleus collapses (138), the DNA content is detected by the cGAS-based surveillance mechanism that links genome instability to innate immune responses (141). Harding et al. showed that cell cycle progression through mitosis following dsDNA breaks induced by 10-20 Gy RT, leads to the formation of micronuclei, which precede activation of inflammatory signaling and are a repository for cGAS (142). For instance, Vanpouille-Box et al. found that cytoplasmic dsDNA was about ten times more abundant in TSA cells exposed to a single dose of 8 Gy or 3 fractions of 8 Gy (X-rays) compared with untreated cells. This was associated with the release of IFN-β and increased expression of IFNAR1 and CXCL10 (143). In addition, it is unclear how cytoplasmic dsDNA is transferred from cancer cells to immune cells, especially to DCs, although transfer *via* exosomes has been suggested (144, 145).

Radiation-induced pro-immunogenic effects in cancer cells are observed in conventional RT with radiation doses from 2 Gy up to 30 Gy or more; however, the optimal radiation regimen to induce a clinically relevant anti-tumor immunity remains to be defined (13, 146). The previous examples about micronucleus and cytosolic dsDNA accumulation suggest a complex relationship between irradiated tumor and host immune system. Vanpouille-Box et al. investigated dsDNA content in the cytosol of cells exposed to radiation (X-rays) doses ranging from 0 Gy to 30 Gy in different murine and human cancer cell lines (143). Surprisingly, they observed that dsDNA accumulates in the cytosol up to a critical threshold when it abruptly decreases at doses between 12 to 18 Gy. The authors demonstrated that doses above this threshold do not confer immunogenicity, mainly due to the dose-dependent upregulation of three-prime repair exonuclease 1 (TREX1). TREX1 is a DNA nuclease with a main role in the degradation of cytoplasmic double- and single-stranded DNA (147). Vanpouille-Box et al. found in TSA cells that upon RT (single doses above 12 Gy), cytosolic dsDNA is cleared by TREX1, precluding the activation of the cGAS pathway to induce type I IFN, therefore abolishing the RT-induced anti-tumor immune response (143, 148).

Cytosolic leakage of mitochondrial DNA (mtDNA) also results in activation of the cGAS–STING pathway (149–151). Mitochondria are sources of ROS that plays a major role in the induction and persistence of oxidative stress following exposure to radiation (152). They are also involved in non-targeted radiation effects (153, 154), suggesting their implication in radiation-induced systemic responses. However, mtDNA is not the primary target of radiation. Friedland et al. used track structure simulations to demonstrate that the probability of DNA double strand breaks induction in mtDNA is about 0.03% at 1 Gy of γ-rays or densely ionizing radiation (155). The involvement of mitochondria in late radiation effects is more likely to be an indirect consequence of ROS generation after irradiation and of the nucleus–mitochondrion signaling pathway. Nevertheless, mtDNA might leak in the cytosol after a direct hit from a charged particle, such as beta particles (e.g. ^177^Lu, ^90^Y radionuclides), alpha particles (external α beam or ^225^Ac/^213^Bi radionuclides), or Auger electrons (e.g. ^125^I).

Altogether, these observations highlights the facts that radiation-induced micronuclei and dsDNA are required for anti-tumor immunity induction *via* cGAS sensing and STING activation. However, the radiation regimen (type of particles, dose, fractions, etc.) to obtain these effects in patients is not known yet. In 2014, a phase II clinical trial was started in patients with NSCLC who progressed after chemotherapy and with at least two measurable disease sites to determine whether radiation and immunotherapy with ipilimumab can stimulate the immune system and stop the growth of tumors that are outside the field of radiation (NCT02221739). Patients receive ipilimumab within 24h of local RT initiation (6 Gy × 5 fractions, 3D-CRT or IMRT). In the case of lack of response, a second phase II trial will be performed with a new RT regimen (9.5 Gy × 3 fractions).

### cGAMP in Bystander Immunity

The role of cGAS and STING in the bystander communication between tumor and non-tumor cells is linked to the concept of cGAMP, a second messenger that activates the STING pathway. Deng et al. demonstrated that exogenous cGAMP treatment promotes the antitumor efficacy of radiation (156). In wild-type mice, the cGAMP and radiation combination reduces tumor burden more effectively than cGAMP or radiation alone. Moreover, about 70% of mice in the combination arm showed complete tumor regression at treatment completion (156). All these data indicate that boosting STING signaling activation can enhance tumor growth inhibition after irradiation. Moreover, Liu et al. demonstrated that in mice grafted with B16-OVA melanoma cells (intravenous injection to model lung metastases), nanoparticle-cGAMP inhalation synergizes with fractionated RT (8 Gy × 3 in the right lung) to generate a potent antitumor immunity against melanoma metastases in both irradiated and non-irradiated lungs (157). This combination led to metastasis growth inhibition in the irradiated and non-irradiated lung, and complete regression of lung metastases in some mice, through TME remodeling (157).

Cytoplasmic cGAMP can diffuse to adjacent cells *via* gap junctions (158, 159). Ablasser and colleagues clearly described a unique immune signaling mechanism that comprises cGAMP production by cGAS in the sensing cell, which is transmitted through gap junctions to bystander cells, leading to remote STING activation and subsequent antiviral immunity. Noteworthy, type I IFN-dependent induction of antiviral immunity in bystander cells takes considerably longer, given the requirement of *de novo* transcription and translation. Therefore, cancer cell-derived cGAMP following irradiation could provide a fast antitumor immune response. These data suggest that bystander activation and signal amplification could have a beneficial role in RT; however, cGAMP transfer might at the same time aggravate cancer resistance and the metastatic potential of STING-dependent tumors. For instance, Chen et al. demonstrated that functional CX43-based gap junctions between cells allow cGAMP transfer from cancer cells to astrocytes (159). This leads to the activation of the STING pathway and the release of cytokines, including IFNα and TNF, which provide a growth advantage to brain metastatic cells by protecting them against physiological and chemotherapeutic stresses. Unlike the transfer of cGAMP to bystander cells that intensifies the immune response, cGAMP transfer from brain metastatic cells to neighboring astrocytes triggers downstream signaling that supports metastatic outgrowth.

Schadt et al. proposed that cGAMP, and not cytoplasmic dsDNA, is transferred from cancer cells to DCs in a CX43-dependent manner, thus enabling the production of type I IFN and antitumor immunity priming (160). This connexin-dependent transfer of cGAMP was corroborated by Pepin et al., who observed the potentiation of macrophages cultured with the conditioned medium of cGAMP producing cells (161). Similarly, Marcus et al. showed that cGAMP, and not dsDNA, is transferred from cancer cells to DCs (162). Indeed, experiments using transplantable tumor models in STING- and cGAS-deficient mice revealed that cGAS expression by tumor cells is critical for tumor rejection by NK cells. Conversely, cGAS expression by host immune cells is not necessarily required, suggesting that tumor-derived cGAMP is transferred to non-tumor cells where it activates STING (162). These observations raise questions about the molecular mechanism involved in the fusion of sEVs purified from tumor cells with recipient (bystander) cells. Indeed, it would be important to know what surface molecules allow their fusion with the recipient cell membrane for cGAMP or dsDNA delivery into the cytosol. Alternatively, other mechanisms could also contribute, such as formation of channels between the apposed membranes of a sEV and the recipient cell (163).

**Figure 1:**
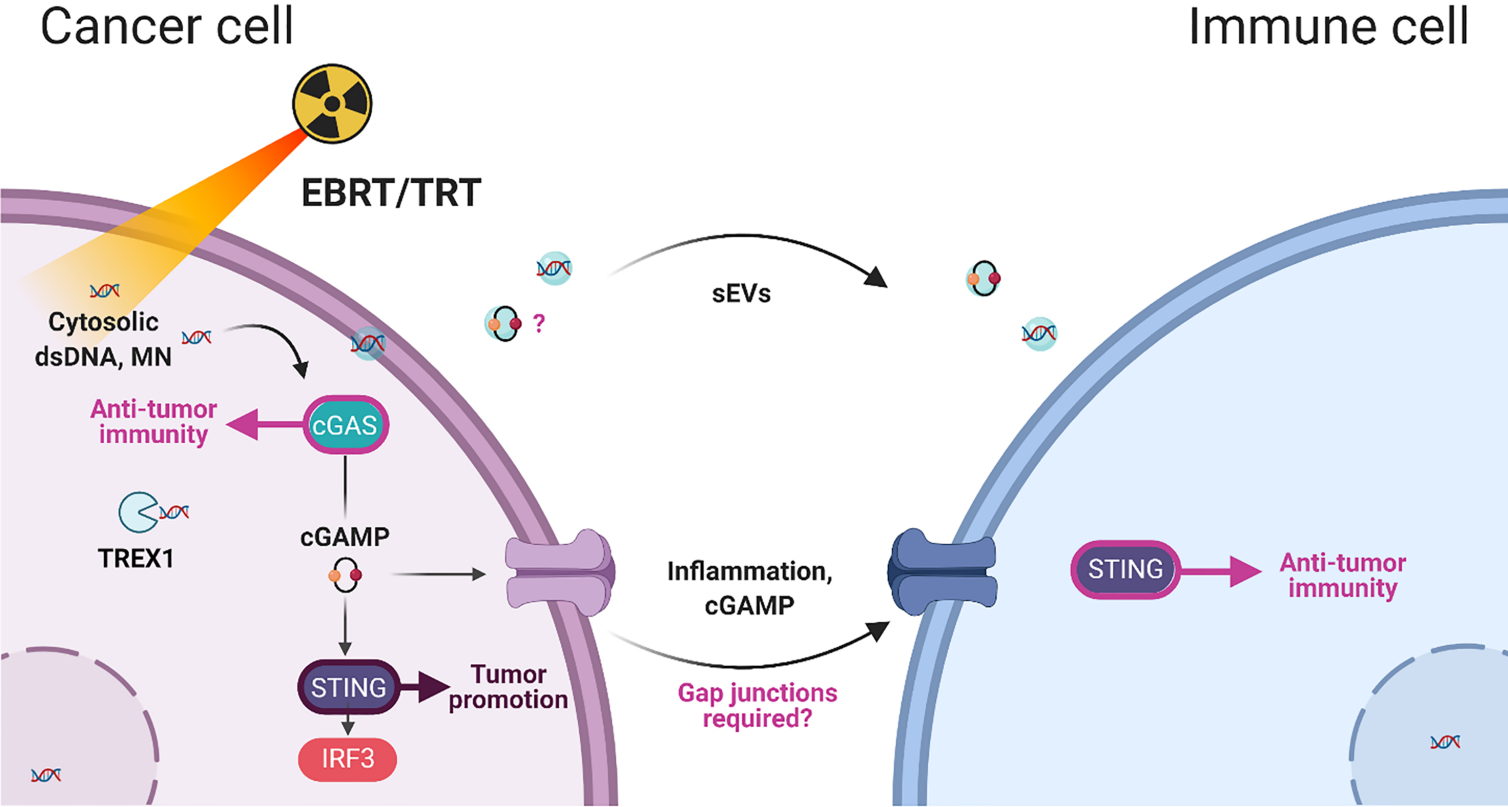
Summary of cancer-immune cell interactions after irradiation (EBRT, external beam radiation therapy; TRT, Targeted Radionuclide Therapy) and the involvement of dsDNA,double-stranded DNA; MN, micronucleus; sEVs, small extracellular vesicles; and cGAMP, cyclic GMP–AMP in bystander immunity.

Overall, these studies demonstrated that cancer cell irradiation leads to cGAMP release in immune cells and that STING has a major role in immune cells in radiation-induced immunity, while it is not required in tumor cells. Furthermore, Bakhoum et al. showed that the cGAS-STING pathway is activated in human cancer cells with chromosomal instability. Improper segregation of chromosomes during cell division leads to the formation of unstable micronuclei, releasing their DNA into the cytosol. In this study, Bakhoum et al. demonstrated that inflammatory response involves activation of NF-κB signaling and promotes metastasis in a STING-dependent manner (164). Accordingly, our recent data suggests that STING expression in lung cancer cells might contribute to tumor formation and that low STING expression in these cells fails to induce type-I IFN expression and potentially favors the establishment of an immunosuppressive microenvironment (165). **Figure 1** summarize the bystander communication between cancer cells and immune cells.

### The STING Pathway in the Induction of the Senescence-Associated Secretory Phenotype and of RT-Induced Adverse Effects

**Figure 2:**
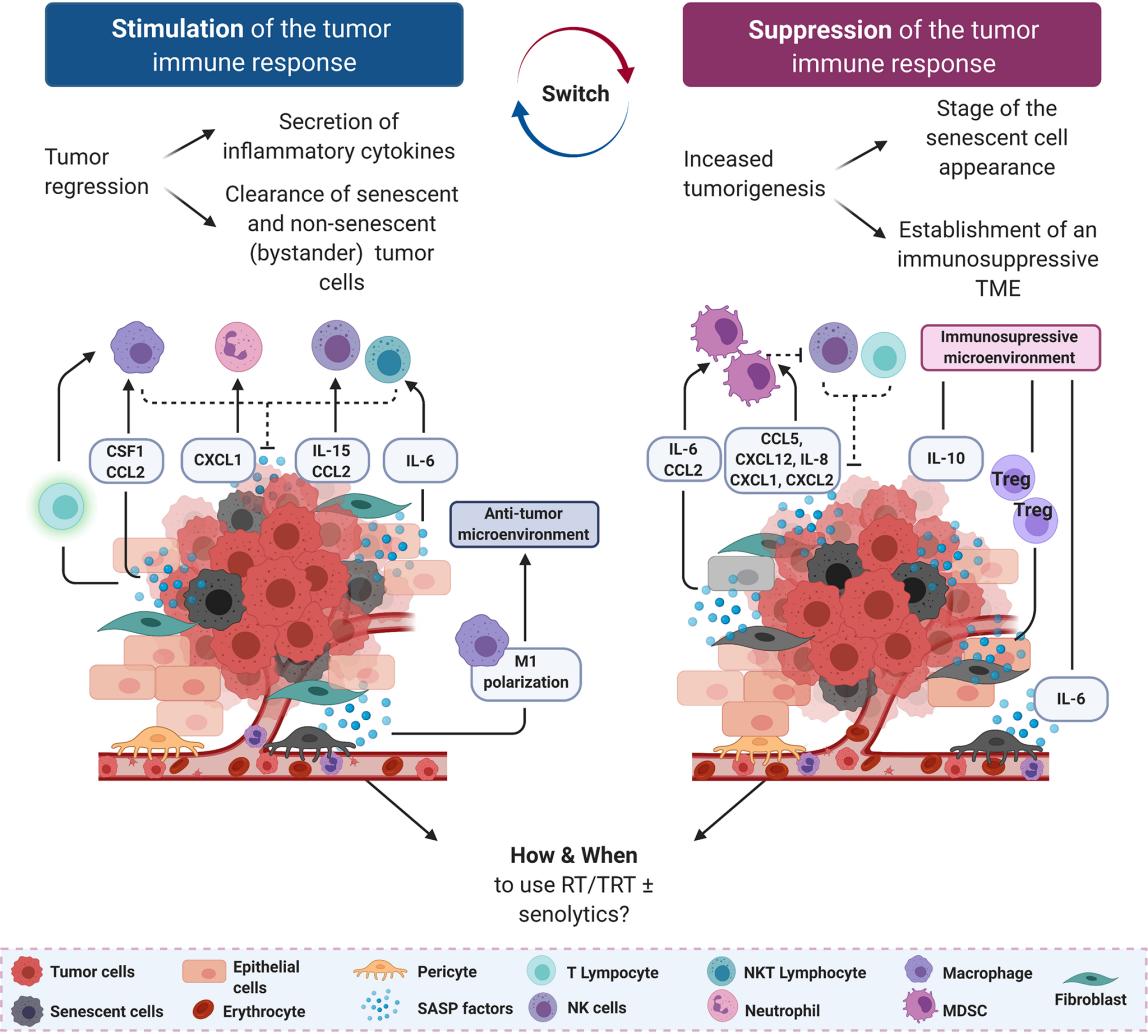
Senescence-associated secretory phenotype (SASP) factors can support or suppress anti-tumor immune responses. On the left, in an immunostimulatory scenario, SASP factors secreted by tumor cells and pericytes drive the recruitment of innate immune cells (macrophages, neutrophils, natural killer (NK) and NK T cells) to mediate the clearance of senescent tumor cells. On the right, in an immunosuppressive scenario, SASP factors secreted mostly by stromal cells recruit immature myeloid cells and myeloid-derived suppressor cells (MDSCs) to dampen the cytotoxic effect of NK cells and CD8+ T lymphocytes. Anti-inflammatory mediators, including IL-6 and IL-8, are also secreted by senescent stromal and tumor cells, further increasing the immunosuppressive environment. Senescent cells are represented by a gray cytoplasm, regardless of their origin. CCL, C–C motif chemokine ligand; CXCL, C–X–C motif chemokine ligand; NK natural killer; NKT, natural killer T lymphocyte.

Through DDR activation, ionizing radiation is a potent driver of accelerated cancer cell senescence, a process that involves ATM, ATR, DNA-dependent protein kinases (166), p53, P16INK4a, p21WAF1, CHEK1 and CHEK2 (167), in breast cancer, colon carcinoma, neuroblastoma and fibrosarcoma. Although senescent cells have exited the cell cycle, they can maintain an active metabolic activity and participate in resistance to therapy and disease progression (168). Indeed, senescent cells can secrete many different bioactive molecules, such as cytokines, proteases and growth factors that influence and shape the surrounding microenvironment. This has been described as Senescence-Associated Secretory Phenotype (SASP) (169). Among the many SASP factors, IL-6, CCL5, CXCL12, CCL2 and IL-8 have a particularly important role in supporting cancer cell metastasis formation and the establishment of an immunosuppressive microenvironment, although in some cancer models they can be found in the immune stimulatory secretome (**Figure 2**).

As RT can induce tumor cell senescence, NK cell recruitment by SASP factors could be a general mechanism by which NK cells help to clear tumor cells in response to senescence-inducing therapies (170). Indeed, in a mouse model of radiation-induced osteosarcoma, the retinoblastoma tumor suppressor gene (RB1) is required for SASP expression and infiltration of NK T-cells in bones of mice exposed to carcinogenic doses of ^45^Ca (four postpartum injections; low energy beta-emitting particles) (171). Il-6 and MIP2 (the murine homolog of IL-8) induce neutrophil accumulation *in vivo* (172), and MIP2 is also implicated in NK T-cell recruitment to the spleen (173). Kansara et al. showed that *Cd1* ^–/–^ mice, lacking NK T cells, are predisposed to ^45^Ca-induced osteosarcoma development when crossed with *Trp53* ^+/–^ mice, consistent with previous findings that NK T-cells play an important role in sarcoma development (174). Growth inhibition of IL-6-deficient osteosarcoma cell lines in wild type mice is accompanied by NK T-cell infiltration, further supporting a role for these cells in host-dependent tumor suppression *in vivo*. Interestingly, in this model, IL-6 not only recruits NK cells that limit tumor growth, but also reinforces the senescence phenotype through autocrine and paracrine mechanisms (171), indicating that bystander (initially) non-senescent tumor cells can be targeted as well (**Figure 2**, left panel). Better understanding how radiation induces SASP factors (dose, fractions, etc.) production by osteoblasts could be beneficial for the management of patients with bone metastases treated with Xofigo™ (^223^Ra, alpha emitting particles) among whom some reported jaw osteonecrosis (175).

Extranuclear DNA sensing *via* the cGAS-STING pathway might play a major role in radiation-induced SASP. The involvement of cGAS in senescence induction has been shown in primary human lung cells (IMR90) in which cGAS and STING knockdowns abolish expression of key SASP-related markers (p16INK4a, IL-8, CXCL1,2,3, IL-6 and CCL2) upon senescence induction with HRasV12 or etoposide. Senescence induction is also reduced in STING knockout mice, as indicated by the absence of hair greying three months after sublethal irradiation, and the impaired immunosurveillance against N-Ras (liver tumor formation) (176). Senescence induction in p53-proficient cells is an important protection mechanism against cell transformation upon oncogenic signaling activation (PTEN loss, Ras signaling). Hence, activation of the cGAS-STING pathway in cells during oncogene-induced SASP is also tightly linked to the expression of the cytoplasmic exonuclease MRE11, TREX1 and DNase2 that rapidly degrade cytoplasmic DNA fragments (177, 178). Whereas DNases mediate the clearance of dsDNA, an excessive amount of DNA escaping from DNases is responsible for induction of type I IFN, through the activation of DNA sensors such as the cGAS-STING pathway. Conversely, cGAS, STING, TBK1 and IRF3 knockdowns are characterized by reduced p21 expression in HeLa cells that leads to higher mitotic activity and ultimately chromosomal instability (179). Altogether, these observations demonstrate that the cGAS-STING pathway might play an important role in maintaining chromosome integrity through senescence induction, and that in this context this pathway also contributes to SASP instauration in cancer cells. However, senescence induction and SASP are intrinsically linked to a functional p53 pathway, and the functionality of the STING-IRF3 pathway in cancer cells harboring p53 mutations has not been investigated yet.

On the other hand, SASP induction following ionizing radiation promotes tissue fibrosis (180). For instance, type-II pneumocyte (181) and alveolar stem cell (182) senescence contributes to RIF in lungs. Similarly, endothelial cell senescence induced by RT is causal in the establishment of cardiovascular disease (183). Considering the critical role of STING signaling in the expression of the complete SASP phenotype, STING expression in endothelial cells and pneumocytes might directly contribute to these RT-induced deleterious effects. *In vitro*, irradiation (2 Gy) of human coronary artery is sufficient to activate the STING pathway and consequently type-I IFN expression (184). Furthermore, STING contributes to cardiac hypertrophy and fibrosis in a model of pressure-overload cardiac hypertrophy through the recruitment of inflammatory macrophages and the release of angiotensin-II (185). STING might also play an important role in the endothelial cell response to RT. Indeed, tumor-derived cGAMP can drive endothelial cell activation, leading to upregulation of adhesion molecules (V-CAM1, I-CAM1) and T-cell recruitment. Constitutive STING activation (due to a mutation) drives microvessel thrombosis and pulmonary syndrome development in infants through an autoimmune reaction, leading to chronic inflammation and macrophage recruitment (186). This reaction that involves endothelial cell dysfunction and chronic sterile inflammation is reminiscent of RT-induced lung fibrosis and maculopathy. All these data suggest that STING signaling in endothelial cells might contribute to the anti-tumor immunity through recruitment of immune cells. However, most of the observation made *in vivo* and in patients suggest that endothelial cell STING signaling could also be an important player in RT-induced cardiac toxicity (187) and possibly lung fibrosis. The impact of STING expression in fibroblasts on RT response remains to be elucidated. Finally, these studies suggest that because many current standard treatments for cancer can induce senescence, which can have wide-ranging effects, some patients might benefit from the addition of senolytic therapy to inhibit the pro-tumorigenic stroma.

Altogether, these observations highlight the key position of STING signaling following RT where it contributes to cancer immunogenicity, DC activation and anti-tumor T-cell response, while simultaneously playing a central role in SASP induction in many cell types. This might be an initiating event towards the aggravation of RT-induced cytotoxicity.

## Concluding Remarks

In the past decade, RT entered the era of personalized medicine, thanks to the striking improvements in radiation delivery, treatment planning optimization, and better understanding of the cancer response. However, the next challenge is to identify the optimal RT regimen to induce a clinically relevant anti-tumor immune response. Indeed, bystander and abscopal effects have been demonstrated in preclinical studies and in some clinical cases, but the exact dose threshold and range need to be defined in function of the tumor type and characteristics, and the patient’s immune status. We hypothesize that radiation could be used as an immunological adjuvant, by lowering the dose per fraction (and/or the total dose) in “hot” tumors, specifically to preserve the viability of intra-tumor lymphocytes. Conversely, the dose could be increased in “cold” tumors. However, healthy organs at risks and the TME often limit the radiation regimen possibilities due to the high risk of adverse toxicities. For instance, radiation reduces the endothelial barrier permeability, facilitating the release of pro-inflammatory factors that orchestrate the architecture of the tumor immune microenvironment. Also, RT-activated macrophages have been repeatedly associated with RT-induced toxicity. Therefore, it is important to find how to modulate macrophage activation to avoid deleterious phenotypes.

RT involves the activation of an anti-tumor response through cytosolic dsDNA sensing by the cGAS-STING pathway. However, a major open question is how to choose the most effective radiation regimen to increase dsDNA accumulation without reaching the critical threshold leading to the activation of DNases, such as TREX1. Interestingly, tumor and immune cells can communicate through the transfer of cGAMP and sEVs, demonstrating that cGAS expression by host immune cells is not necessarily required, while STING is.

Furthermore, there is still an important gap of knowledge on the cGAS-STING pathway role in cancer cell SASP induction upon RT. STING signaling following RT contributes to cancer immunogenicity, DC activation and anti-tumor T-cell response, while simultaneously playing a central role in SASP induction in many cell types. SASP induction is involved and most probably is an initiating event in the aggravation of many RT-induced cytotoxicity events. Radiation dose threshold and SASP are linked through the expression of cytoplasmic DNases, such as TREX1. Once again, the fine-tuning of radiation dose regimens should allow an optimal anti-tumor immune response while limiting adverse effects.

## Outstanding Questions

- What are the optimal radiation dose regimens and fractions?
- What is the best therapeutic window to enhance RT anti-tumor immune response?
- How is the cGAS-STING pathway playing an important role in cancer cell SASP induction upon RT?
- What is the impact of STING expression in fibroblasts exposed to radiation?
- In which conditions inhibitors (e.g. ATM, STING) should be concomitantly administered with RT?
- Whether and when, during cancer development, a senolytic treatment or a drug targeting the SASP should be employed?

## Author Contributions

The review results from the discussion and the consensus of all authors listed (JC, JF, CU, CB, and J-PP). Literature review on the topic was analyzed, produced and written by JC, JF, CU, CB, and J-PP. All authors contributed to the article and approved the submitted version.

## Funding

This work was supported by SIRIC Montpellier Cancer Grant INCa_Inserm_DGOS_12553, by INCa-Cancéropôle GSO, by AVIESAN PCSI [grant number : ASC20025FSA].

## Conflict of Interest

The authors declare that the research was conducted in the absence of any commercial or financial relationships that could be construed as a potential conflict of interest.
